# Characteristics of Brazilian dental malpractice lawsuits in 2022 and 2023

**DOI:** 10.5281/zenodo.16418708

**Published:** 2025-08-01

**Authors:** Flavia Vanessa Greb Fugiwara, Patricia Nakasato Kondo, Deisy Satie Moritsugui, Rodolfo Francisco Haltenhoff Melani

**Affiliations:** 1 Department of Community Dentistry, School of Dentistry, University of São Paulo, São Paulo, SP, Brazil.

**Keywords:** Malpractice;, Forensic Dentistry,, Legal Liability;, Case Law

## Abstract

A survey was conducted of the dental professional liability cases adjudicated (post-appeal) throughout the Brazilian territory during the years 2022 and 2023. Data was retrieved from the Jusbrasil website (www.jusbrasil.com.br), and the characteristics of the lawsuits were assessed and compared with those reported in previous studies. The query entered into the “case law search” field was “dental professional liability”. An assessment was conducted of 472 rulings referring to 2022 and 447 rulings referring to 2023 in which the dentist was convicted in 61% and 68% of the cases, respectively. There was no increase in the number of cases between the years included in the assessment; however, an increase did occur in the number of cases when compared to that reported in another methodologically similar study. An expert’s report was solicited by the court in 75% of the cases. The most frequently involved specialties were implant dentistry and prosthodontics, and there was a tendency toward an increase in lawsuits involving companies as opposed to individuals. The amounts awarded for moral and aesthetic damages ranged from US$ 299.00 to US$ 19,960.00. The highest mean value awarded for moral damages involved the specialty of oral & maxillofacial surgery and traumatology, whereas the lowest mean value was associated with restorative dentistry. The amount most frequently awarded in convictions across the two years included in the survey was US$ 1,954.00. The adoption of preventive measures can potentially reduce the number of new cases, convictions and financial losses resulting from civil liability lawsuits.

## INTRODUCTION

For many years, dental practice primarily focused on diagnosing, preventing, and treating oral diseases. However, over the last decade, its scope has expanded and now carries a strong emphasis on ethical conduct, patients' right to information, and the meticulous documentation of clinical cases.

There has been an increasing emphasis and societal recognition of clinical outcomes, especially concerning their aesthetic aspect. While this aspect predominantly relies on subjective perception, it significantly influences patients' overall assessment of their treatment outcomes.

Professionals, influencers, and celebrities often post impressive dental treatment results on social media without considering the technical limitations or even patient limitations involved, creating beauty standard expectations that are often unattainable.

When dentists fail to adequately explain treatment limitations, patients may become frustrated with unmet expectations regarding treatment outcomes, potentially leading to dissatisfaction. Consequently, patients may resort to legal action in an attempt to resolve disputes arising from perceived shortcomings in their treatment.

Health professionals may encounter adversities during the procedures they perform, which may or may not result in harm to patients. According to Article 927 of the Brazilian Civil Code of 2002, “Whoever causes damage to others by an unlawful act (Art. 186 and 187) is obliged to provide reparation for it.” Furthermore, article 186 stipulates that “Anyone who violates the rights of others or causes them harm through voluntary action or omission, negligence, or imprudence commits an unlawful act, even if said harm is exclusively moral in nature.” (*1*) Therefore, unless exculpatory circumstances are present, civil liability arises whenever harm is inflicted upon a patient due to culpable conduct on the part of the dentist and a causal link can be established between the harm and the conduct.

Civil liability aims to restore the moral and financial equilibrium that may have been disrupted due to the actions of a professional, thereby imposing an obligation to compensate (*2*) the injured party. This compensation serves the purpose of providing reparation for financial losses and/or for the infringement of the injured party's honor or image, thus characterizing harm to their physical integrity that could lead to embarrassment. (*3*)

Regional studies conducted over the past six years indicate that the dental specialties most commonly implicated in lawsuits were implant dentistry, prosthodontics, orthodontics, oral & maxillofacial surgery and traumatology, and endodontics. (*4*–*9*)These findings are consistent with nationwide surveys that analyzed data retrieved from the websites of State Courts of Justice spanning from 1974 to 2006, (*10*) from January 2006 to August 2011, (*11*) as well as data obtained from the Jusbrasil website pertaining to the year 2017. (*12*)

The aim of this study was to evaluate the features of nationwide cases adjudicated in 2022 and 2023 pertaining to dental professional liability. This assessment was conducted through a search on the Jusbrasil website, and the results obtained were compared with findings from previous studies.

## MATERIALS AND METHODS

This cross-sectional study gathered data on dental malpractice lawsuits from the publicly accessible Jusbrasil website (www.jusbrasil.com.br). The collected information encompassed lawsuits published between January 1, 2022, and December 31, 2023.

The search included decisions handed down after appeal concerning allegations of harm filed against individuals and/or companies across all State Courts of Justice in Brazil. The search query utilized in the “case law search” field was “dental professional liability.”

The data collected by two researchers regarding the rulings assessed (appellate court panel decisions) included the following variables: state of the federation, date of publication, defendant (individual, clinic, private health insurer, educational institution, hospital, local government, or franchisor), dental specialty involved, trial court and appellate court decisions, expert opinion, and compensation amounts (if any) awarded for moral and/or aesthetic damages.

The exclusion criteria comprised cases involving refunds for treatments that were not administered, since the merit in these cases was not based on damage caused by the dental procedure itself. Additionally, cases returned to the first instance trial court and those redistributed to other courts were also excluded from the analysis.

Compensation amounts not explicitly stated in the ruling, as well as those based on the number of minimum wages or involving combined aesthetic and moral damages, were excluded from consideration.

The specialties involved were categorized by the researchers according to the information provided in the appellate court rulings, rather than relying on the classifications used within the decisions. This approach acknowledges the potential for inaccuracies in technical classifications employed by judicial bodies.

The specialties of orthodontics and functional jaw orthopedics were consolidated into a single category. Procedures for installing veneers and dental contact lenses were categorized under restorative dentistry. Bone grafting procedures conducted in preparation for implant installation were attributed to implant dentistry, while tooth extractions were classified within oral & maxillofacial surgery and traumatology. The data were then tabulated and analyzed utilizing a Microsoft Excel spreadsheet.

## RESULTS

Information from 472 decisions in 2022 and 447 decisions in 2023 were assessed, resulting in a total of 919 rulings. The majority of these rulings originated from the state of São Paulo, followed by the state of Rio de Janeiro (Table 1).

**Table 1:** Rulings of 2022 and 2023 according to Brazilian state

**State**	**Number of rulings**	**Number of rulings**
**2022**	**2023**
São Paulo (SP)	172	145
Rio de Janeiro (RJ)	71	84
Rio Grande do SUL (RS)	50	25
Minas Gerais (MG)	47	50
Paraná	40	28
Federal District (DF)	31	25
Santa Catarina (SC)	25	19
Other	36	71
Total	472	447

As for individually involved defendants, a higher number of dentists (individuals) and clinics were observed (Table 2). When considering joint involvements, individuals were defendants in 275 cases in 2022 (58%) and 233 cases in 2023 (52%). Clinics were defendants in 265 cases in 2022 (56%) and 281 cases in 2023 (63%). However, when considering all companies involved, they were defendants in 313 cases in 2022 (66%) and 327 cases in 2023 (73%).

**Table 2:** Defendants involved in the rulings

**Defendant**	**2022**	**2023**
Individual	156	114
Clinic	153	173
Clinic and dentist	92	93
Educational institution	14	11
Private health insurer and dentist	10	11
Clinic, dentist and private health insurer	9	5
City/State government	8	6
Private health insurer	7	10
Private health insurer and clinic	7	2
Franchisor and clinic	4	5
Other	12	17
Total	472	447

Table 3 shows the number and percentage of rulings, whether a technical expert’s report was commissioned, and the number and percentage of convictions for the two years included in the survey according to the seven specialties most frequently involved. Out of the 919 rulings scrutinized, convictions were recorded in 61% of cases in 2022 and in 68% of cases in 2023. Among the convictions in 2022, 250 (87%) involved compensation for moral damages, and 23 (8%), for aesthetic damages. In 2023, the corresponding figures were 282 (93%) and 28 (9%), respectively. Table 4 shows the average, minimum, and maximum amounts awarded for moral and aesthetic damages, categorized by specialty involved.

**Table 3:** Numbers of rulings, technical expert’s report, and number of convictions according to specialty involved

**Specialties**	**2022**							**2023**						
**Rulings**		Expert’s report			**Convictions**		**Rulings**		Expert’s report			**Convictions**	
**N**	**%**	**Yes**	**No**	**Not reported**	**N**	**%**	**N**	**%**	**Yes**	**No**	**Not reported**	**N**	**%**
Implant dentistry	133	28	109	15	9	95	71	97	22	77	15	5	68	70
Prosthodontics	102	22	68	16	18	66	65	117	26	85	18	14	81	69
Oral & maxillofacial surgery and traumatology	64	14	49	8	7	41	64	75	17	58	9	8	44	59
Orthodontics	53	11	44	4	5	26	49	36	8	31	2	3	27	75
Endodontics	51	11	37	4	10	28	55	46	10	31	7	8	28	61
Orofacial harmonization	12	3	11		1	3	25	5	1	3		2	3	60
Restorative dentistry	11	2	6	3	2	6	55	15	3	11	1	3	12	80

**Table 4:** Amounts awarded (in US$) in convictions for moral and aesthetic damages, according to specialty involved

**Specialties**	**Number of cases**		**Amounts awarded for moral damages**						**Amounts awarded for aesthetic damages**					
**2022**	**2023**	**2022**			**2023**			**2022**			**2023**		
**N**	**N**	**Min.**	**Max.**	**Mean**	**Min.**	**Max.**	**Mean**	**Min.**	**Max.**	**Mean**	**Min.**	**Max.**	**Mean**
Implant dentistry	133	97	573	9560	2284*	399	15968	2435*	382	4780	1584	399	9980	2107
Prosthodontics	102	117	382	8413	1600*	299	15968	486*	1147	1912	1656	399	1596	848
Oral & maxillofacial surgery and traumatology	64	75	764	9560	3096*	798	19960	3165*	956	956	956	998	5988	2661
Orthodontics	53	36	382	4780	2059*	798	11976	1989*	956	956	956	1197	9980	4391
Endodontics	51	46	382	5736	1867	399	5988	1988*	1912	1912	1912	998	1996	1663
Orofacial harmonization	12	5	1147	4780	2613	2794	2994	2594	764	764	764			
Restorative dentistry	11	15	573	2294	1657*	798	2395	1506	956	1912	956			

*Mode amounts of US$ 1,996 (2022) and US$ 1,912 (2023). Average commercial dollar exchange rate in 2022: R$ 5.23. Average commercial dollar exchange rate in 2023: R$ 5.01

## DISCUSSION

The rapid integration of new technologies and resources into the field of dentistry in recent years has brought about various impacts. These advancements present dentists with a significant challenge, as they necessitate ongoing investments of time and resources in continuing education to effectively incorporate them into daily practice. Furthermore, this challenge is compounded by the heightened risk of patients initiating lawsuits against dentists, prompting the adoption of precautionary measures such as acquiring civil liability insurance. (*13*)

These factors have been highlighted by the gradual increase in civil liability lawsuits involving dentists over the years. (*4*–*7*, *9*, *10*, *14*)This escalation may be attributed to several factors: (i) enactment of the Consumer Protection Code (Federal Law No. 8,078/1990), which, although not directly pertinent to healthcare, situates dentistry within the broader context of consumer relations; (*15*) (ii) the massification of dentistry; (*16*) (iii) the increased accessibility to information pertaining to potential harm experienced by patients, facilitated by greater internet accessibility; (*14*) and (iv) the inherent challenges in the patient-professional interpersonal relationship. (*17*) A survey conducted within the orthodontics specialty revealed that the primary reason underlying the grievances (*18*) was dissatisfaction directly linked to the failure to meet patient expectations. (*19*)

Another notable trend is the increase in the provision of free legal counsel, which peaked in 2022, marking the year with the highest number of plaintiffs benefiting from this provision since 2017. (*20*) Through this provision, the payment of court costs and expenses, across all instances, as well as attorney fees for the prevailing party (to be borne by the losing party), are suspended, thus encouraging the filing of lawsuits and appeals to higher courts. (*20*) The processing fee for appeal preparation in the state of São Paulo in 2023 amounted to 4% of the case value, with limits set at a minimum of US$ 34.19 and a maximum of US$ 20,515.00. (*21*) In other words, in a lawsuit valued at US$ 10,000.00, the attorney would be required to pay US$ 400.00 to file an appeal in an appellate court, unless free legal counsel had been granted.

The present study did not identify an increase in the number of civil liability cases in Brazil between the years surveyed, despite such an upward trend being noted in other, (*4*–*10*) predominantly regional studies. (*4*–*7*, *9*, *11*) Nationwide studies, which examined cases up to 2012 by accessing the websites of State Courts of Justice (*10*, *11*) may suffer from incompleteness, given that the digitization of legal cases was only implemented in September of 2009. Although no significant disparity between the evaluated years was found, the number of cases was quite significant. To provide context within the international landscape, it is noteworthy that the city of Rome (Italy) experienced a decline in the number of lawsuits filed against dental service providers. (*22*)

This phenomenon could potentially be attributed to heightened caution among professionals regarding their patients, and to an increase in extrajudicial settlements. (*22*)

Lyra et al. conducted a nationwide study focusing on rulings from 2017 (*12*) by utilizing the Jusbrasil website and employing the search query “civil liability and dentists.” We adopted the same platform in the present study due to the absence of standardized search systems across the websites of State Courts of Justice, which would otherwise impede efforts to establish methodological consistency and reproducibility.

Apart from the investigation carried out by Paula (2007), (*10*) which noted a higher number of cases in the state of Rio de Janeiro, the remaining studies, including the present one, indicated a higher prevalence of cases in the state of São Paulo. São Paulo is the most populous state in Brazil, home to over 44 million inhabitants (22% of the national population), followed by Minas Gerais (10%) and Rio de Janeiro (8%). Therefore, the high incidence of legal cases in this state could be explained by its higher number of inhabitants, along with its higher number of active registered dentists, totaling 114,142 (27% of active professionals), followed by Minas Gerais, with 51,227 (12%), and Rio de Janeiro, with 37,411 (9%).

An inversion is observed when analyzing the defendants involved in the lawsuits, i.e. a reduction in the number of rulings involving individual dentists and an increase in the number of rulings involving dental clinics. This change may be linked to an increase in dental clinic registrations in the country, which quadrupled between 2015 and 2023. (*23*) In contrast, DiLorenzo et al. found that individual dentists were involved in most cases in the Campania region (Italy). (*24*)

In the 2022 rulings analyzed, the specialty most commonly implicated was implant dentistry (28%), followed by prosthodontics (21%), oral & maxillofacial surgery and traumatology (14%), orthodontics (11%), and endodontics (also 11%). In contrast, in 2023, the most frequently involved specialty was prosthodontics (26%), followed by implant dentistry (22%), oral & maxillofacial surgery and traumatology (17%), orthodontics (8%), and endodontics (10%).

Similar results were found in countries that, like Brazil, adhere to the Roman Germanic legal system (civil law), wherein written statutes serve as the primary source of law. Studies conducted in Italy (*22*) and Peru indicated that prosthodontics was the most frequently involved specialty, (*25*) whereas other studies have found that prosthodontics and implant dentistry were the specialties most commonly involved in the lawsuits. (*24*) A study conducted in Iran evaluated 64 rulings related to dental malpractice and observed that fixed prosthodontics was the specialty most frequently involved, followed by oral surgery. Another study conducted in the city of Tehran with 412 rulings arrived at the same conclusion.

In Brazil, implant dentistry was incorporated into the list of dental specialties in 1990. Possibly for this reason, it ranked fourth among the specialties most frequently involved in legal disputes by 2007. (*10*) By 2012, it had moved up to the top position, (*11*) and, thereafter, has consistently featured among the top three specialties most frequently involved in lawsuits. (*4*–*9*, *11*, *12*, *26*) Moreover, a significant number of cases examined in the present study involving the specialty of prosthodontics were found to be associated with harm stemming from treatment procedures involving implant-supported prostheses.

Another aspect evaluated in the study was the appointment of a judicial expert during trial court proceedings to aid the judge in rendering a decision. The analysis revealed that a technical expert's report was included in 75% of the lawsuits filed in 2022 and 2023. In 2022, 55 cases (11.6%) lacked an expert's report, with the dentist being convicted in 37 of them (67%). Similarly, in 2023, 59 cases (13.2%) had no expert's report, with the dentist being convicted in 37 of these cases (62%). In the remaining instances, this information was either ambiguous or not reported in the ruling. These figures stand in contrast to those documented in a previous study, which indicated the complete absence of an expert's report in 34% of the cases. (*9*) Consequently, it is plausible to assert that the absence of an expert's report does not inherently correlate with the conviction of the dentist. Thus, it becomes imperative to evaluate other evidence presented in the case, such as the existence of clinical records.

With regards to the conviction of dentists, Table 5 presents the findings of the present study juxtaposed with those of the research conducted by Loreto et al. (2023).

**Table 5:** Comparison of convictions: present study vs. Loreto et al. (2023)

	**Loreto et al. ^28^**	**Present study**	
Search source	State Court of justice	Jusbrasil	
Scope	Regional	National	
Period surveyed	2003 to 2019	2022	2023
Total rulings	171	472	447
Lawsuits in which the dentist was convicted	68 (40%)	288 (61%)	302 (68%)
Dentist convictions for moral damages	63 (93%)	250 (87%)	282 (93%)
Dentist convictions for aesthetic damages	4 (6%)	23 (8%)	28 (9%)

A survey in Peru found that the most frequently involved specialties were prothodontics, orthodontics, oral surgery, and implant dentistry, at equal rates. However, the conviction rate it found neared 85%, which was higher than the rates found in the present study (61% and 68%). This fact may be explained by the period covered in the Peruvian survey (2011-2016), as well as by an increased demand for implant treatments and broader access to knowledge about implants in Peru. A study carried out in Italy observed a conviction rate of 74%. (*13*), closer to but still higher than that found in the present study.

The assessment of compensation amounts is predicated on the distinction between material damage, which affects an asset with ascertainable economic value, and moral damage, which defies immediate quantification. The present study included figures for both moral and aesthetic damages.

Zanin et al. (2016) observed that compensation amounts for moral damages ranged approximately from US$ 1,000.00 to US$ 30,000.00. (*17*) The minimum amount found by these authors was higher than that found in the present study but lower than that reported by Loreto et al. (*26*) In contrast, the maximum amount recorded in 2016 was the lowest among the three studies. Notably, the present study revealed that the highest average compensation amount was associated with the specialty of oral & maxillofacial surgery and traumatology in 2022 (US$ 3,096.00), whereas the lowest average amount pertained to the specialty of restorative dentistry in 2023 (US$ 998.00). Additionally, it was found that US$ 1,912.00 (2022) and US$ 1,996.00 (2023) emerged as the most frequently awarded amount for moral damages over the two years covered by the survey.

Both aesthetic damages and moral damages entail non-pecuniary compensation and are predicated on the presence of an external injury causing subjective distress and/or social rejection. One plausible definition of aesthetic damage could be understood as “any enduring or permanent alteration in an individual's external appearance;” a change, whether discernible or not, that is irreversible and detrimental. (*3*) In cases of professional liability, the plaintiff may seek compensation for both material damages and moral and aesthetic damages within the same lawsuit, as evidenced in Digest #37 of the Superior Court of Justice (STJ): “Compensation for material damage and moral damage resulting from the same incident may be aggregated.” Additionally, STJ Digest #387 of January 9, 2009 confirms the admissibility of combining compensation for moral and aesthetic damages.

Table 5 shows a rise in convictions for aesthetic damages, which increased from 4 (6%) (*26*) to 28 (9%), indicating a lesser frequency of such claims up to 2019. This trend could be attributed to the 2002 Civil Code, which strengthened the possibility of cumulative aesthetic and moral damages. (*3*) In 2022, the highest amount awarded for aesthetic damages, which occurred more than once, was US$ 1,912.00. Each of the remaining amounts (US$ 2,294.00, US$ 3,824.00, US$ 4,780.00 and US$ 19,120.00) was awarded in only one ruling. In 2023, the sum of US$ 9,980.00 was observed in more than one case.

## CONCLUSION

Based on the findings of the present study, it was concluded that the amounts awarded by courts for moral and aesthetic damages in civil liability cases were substantial during both 2022 and 2023. However, there was no significant difference between these years in terms of the number of cases recorded. Continuing technical updating of dentists, humanized care, and adjusting the patient's expectations to treatment limitations can contribute to a reduction in the number of new lawsuits. Furthermore, Brazilian dentists ought to systematically document both the procedures they perform and their patients’ history to ensure they have the necessary documentation to substantiate the appropriateness of their clinical management decisions. Finally, taking out civil liability insurance can mitigate the risk of sustaining substantial financial losses resulting from adverse judgments in civil liability litigation.
